# Supplementary material for: Exploring the link between molecular cloud ices and chondritic organic matter in laboratory
Source: Nat Commun. 2021 Jun 10;12:3538. doi: 10.1038/s41467-021-23895-2 (PMC8192538; doi:10.1038/s41467-021-23895-2)
Supplement: Supplementary file 1 — Supplementary Information [file 41467_2021_23895_MOESM1_ESM.pdf]

**Supplementary Information: Exploring the link between molecular cloud ices and chondritic organic matter in laboratory**

G. Danger [1,2,3,*], V. Vinogradoff [1,2,*], M. Matzka [4,5], J-C. Viennet [6], L. Remusat [6], S. Bernard [6], A. Ruf [1], L. Le Sergeant d'Hendecourt [1,2], P. Schmitt-Kopplin [4,5]

[1] Aix-Marseille Université, Laboratoire de Physique des Interactions Ioniques et Moléculaires, UMR 7345, CNRS, Marseille, France

[2] Aix Marseille Université, CNRS, CNES, LAM, Marseille, France

[3] Institut Universitaire de France (IUF)

[4] Helmholtz Zentrum München, Analytical BioGeoChemistry, Neuherberg, Germany

[5] Technische Universität München, Chair of Analytical Food Chemistry, Freising-Weihenstephan, Germany

[6] Muséum National d'Histoire Naturelle, Sorbonne Université, UMR CNRS 7590, Institut de minéralogie, de physique des matériaux et de cosmochimie, Paris, France

Corresponding authors: Grégoire Danger and Vassilissa Vinogradoff
**Email:** gregoire.danger@univ-amu.fr and vassilissa.vinogradoff@univ-amu.fr

**Evolution of DBE as a function of m/z for pre-accretional organic residue and post-aqueous organic products**

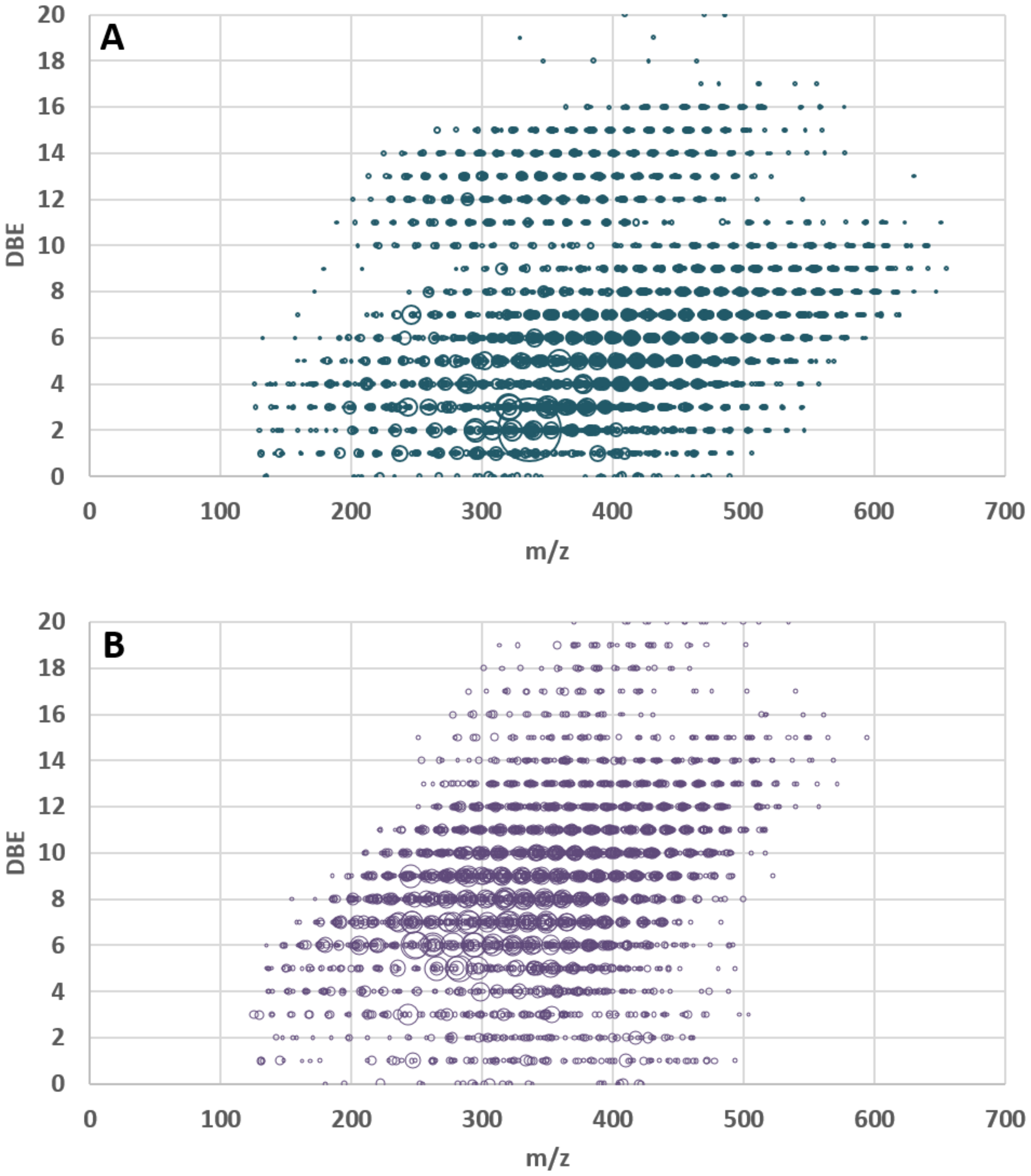


**Supplementary Figure 1 –** Evolution of Double Bound Equivalent (DBE) as a function of the experimental mass (m/z) for the pre-accretional organic residue (3964 molecular attributions) (A), and of post-aqueous organic product after 100 days (3424 molecular attributions) at 150°C (B). The size of the circle representing each molecular attribution is proportional to ion intensities.

**Complementary data regarding the Van Krevelen diagrams for H/C vs O/C of the CHNO family for pre-accretional and post-aqueous organic residues after 100 days of reaction**

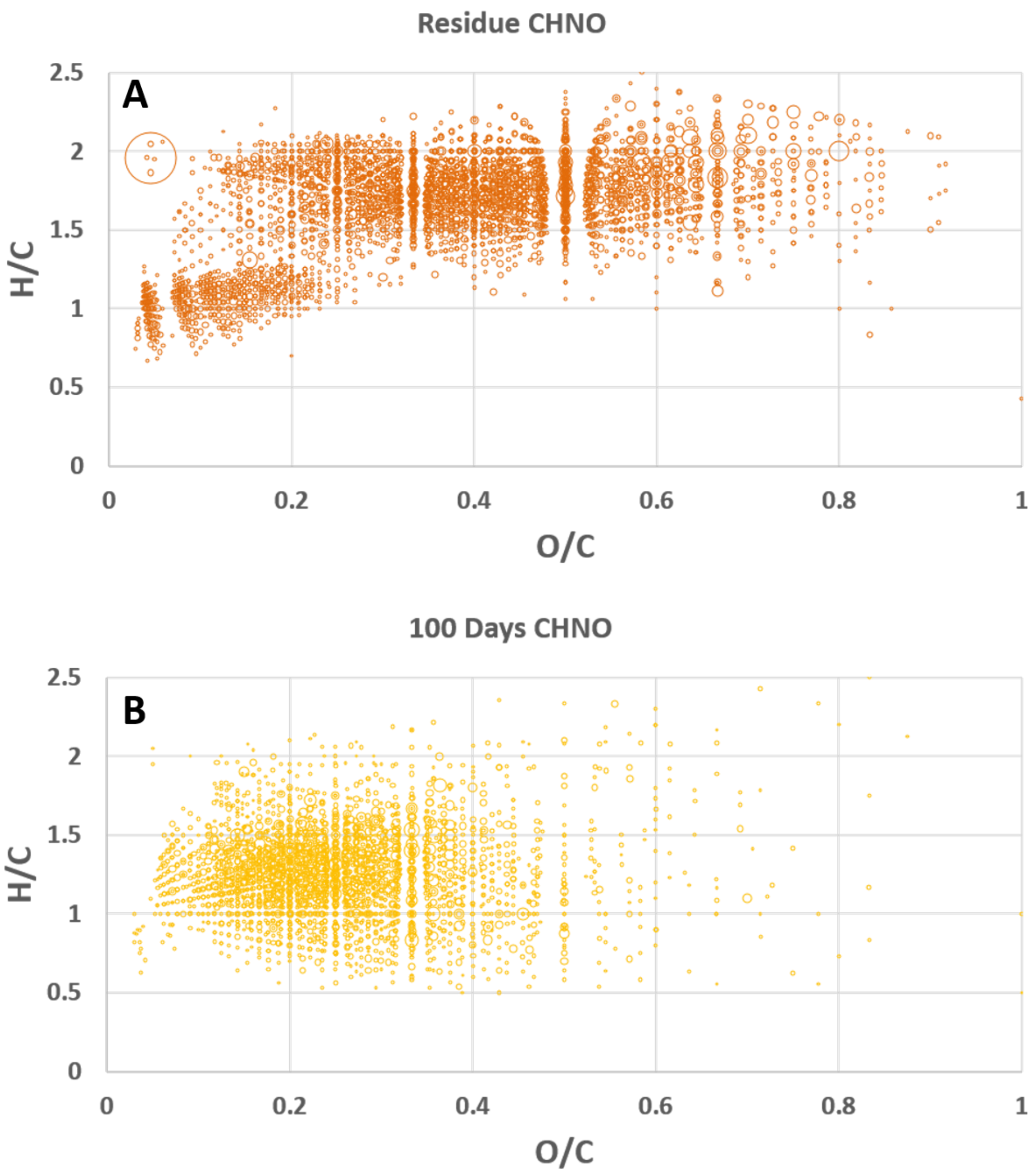


**Supplementary Figure 2–** Van Krevelen diagrams are displayed for H/C vs O/C relative to the CHNO family for pre-accretional (3512 molecular attributions) (A) and post-aqueous organic products after 100 days (2591 molecular attributions) at 150°C (B). The size of the circle representing each molecular attribution is proportional to ion intensities.

**Complementary data regarding the DBE vs O/C of the CHNO family for pre-accretional and post-aqueous organic residues after 100 days of reaction**

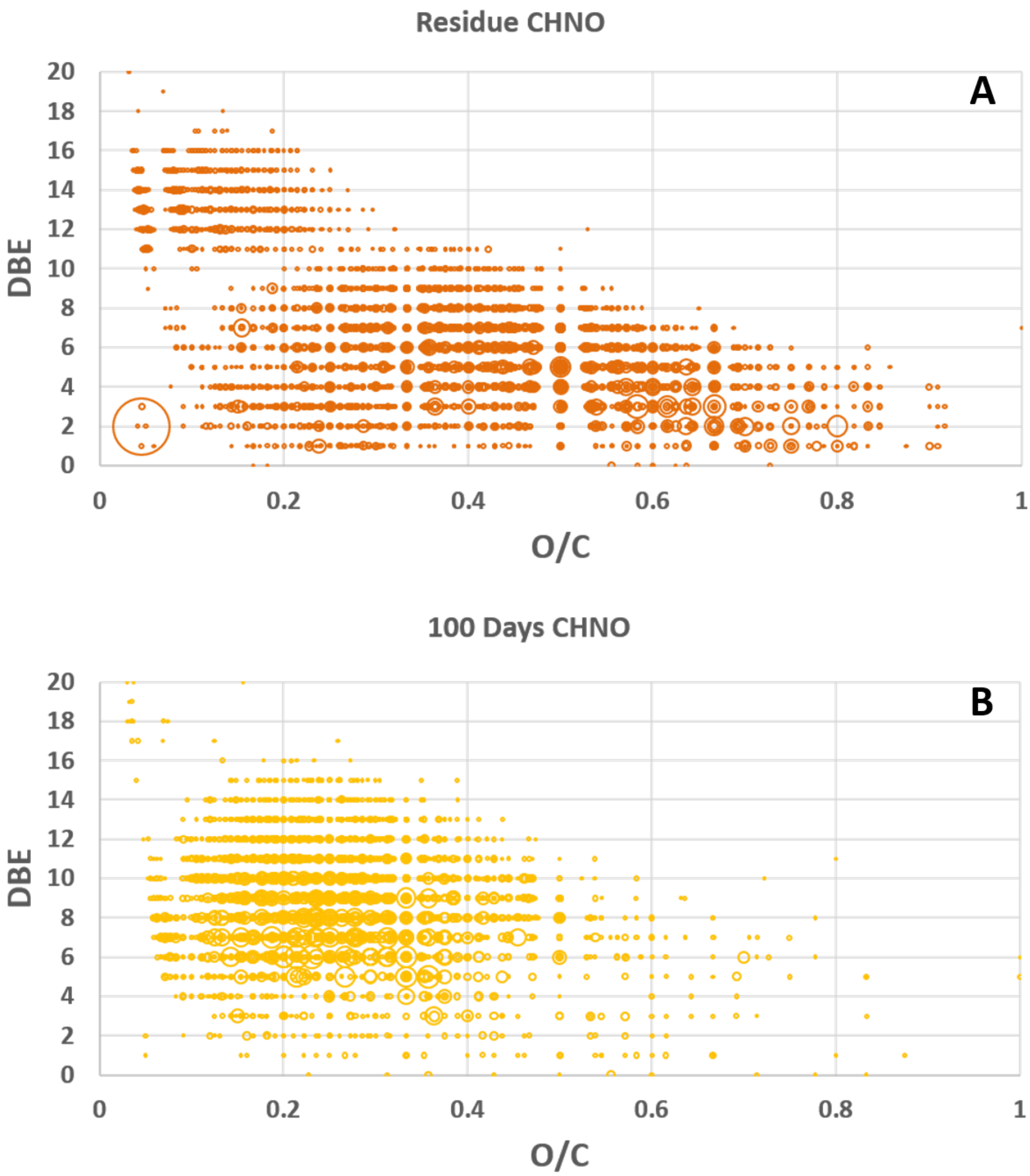


**Supplementary Figure 3– –** DBE vs O/C relative to the CHNO family for pre-accretional (3512 molecular attributions) (A) and post-aqueous organic products after 100 days (2591 molecular attributions) at 150°C (B). The size of the circle representing each molecular attribution is proportional to ion intensities.

**Complementary data regarding the O vs m/z of the CHNO family for pre-accretional and post-aqueous organic residues after 100 days of reaction**

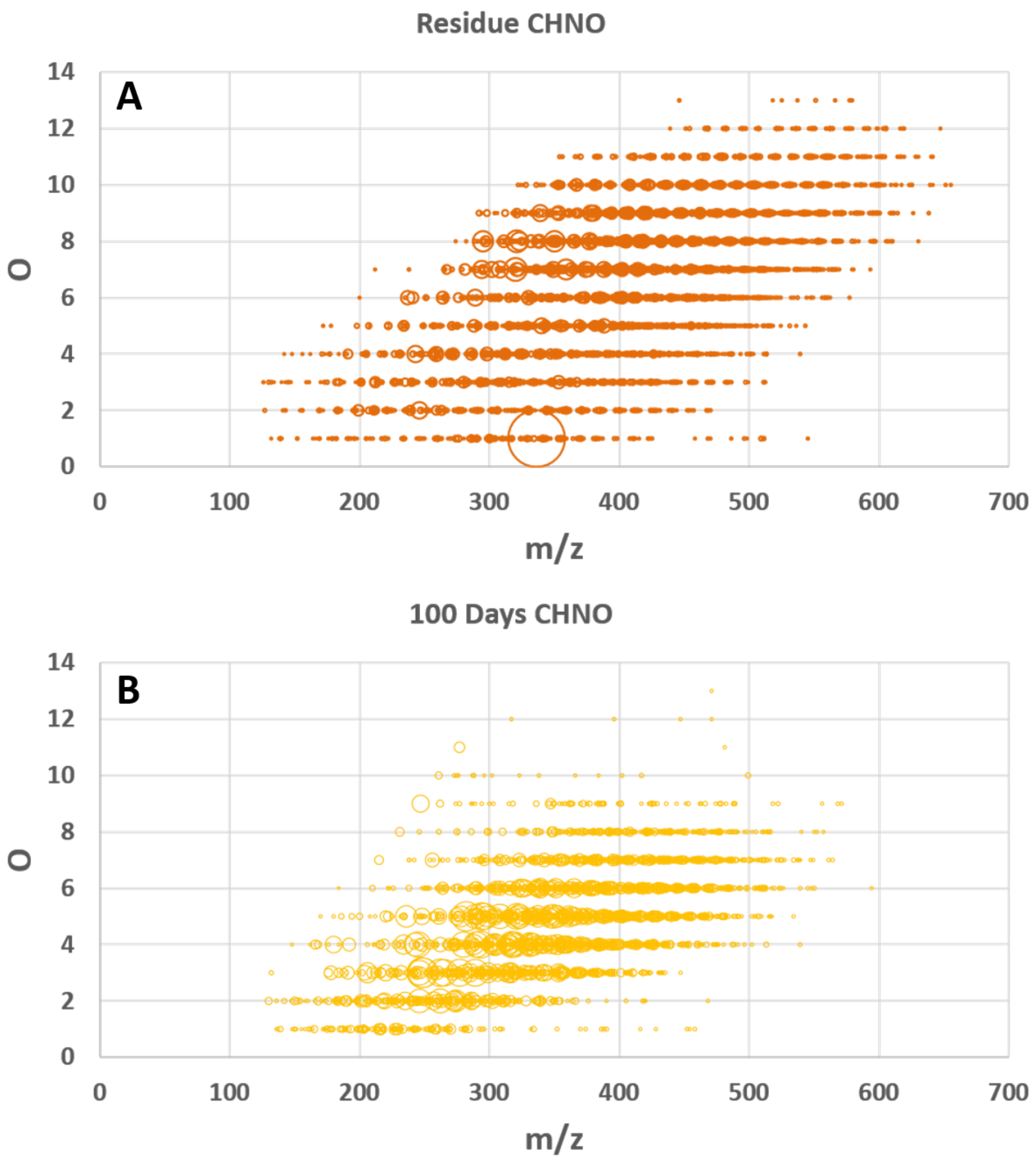


**Supplementary Figure 4– –** O vs m/z relative to the CHNO family for pre-accretional (3512 molecular attributions) (A) and post-aqueous organic products after 100 days (2591 molecular attributions) at 150°C (B). The size of the circle representing each molecular attribution is proportional to ion intensities.

**Complementary data regarding the Van Krevelen diagrams for H/C vs N/C of the CHNO family for pre-accretional and post-aqueous organic residues after 100 days of reaction**

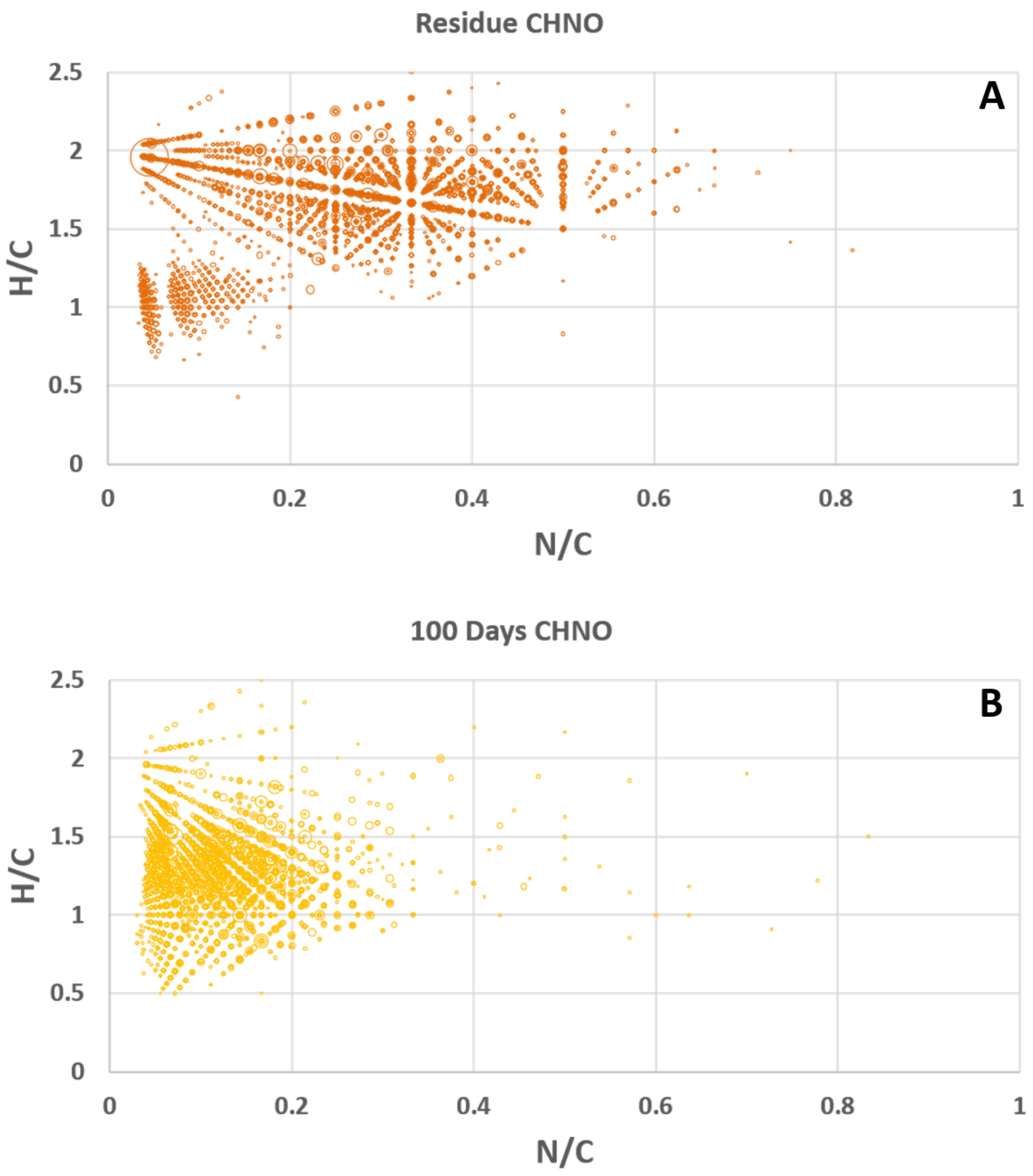


**Supplementary Figure 5–** Van Krevelen diagrams are displayed for H/C vs N/C relative to the CHNO family for pre-accretional (3512 molecular attributions) (A) and post-aqueous organic products after 100 days (2591 molecular attributions) at 150°C (B). The size of the circle representing each molecular attribution is proportional to ion intensities.

**Complementary data regarding the DBE vs N/C of the CHNO family for pre-accretional and post-aqueous organic residues after 100 days of reaction**

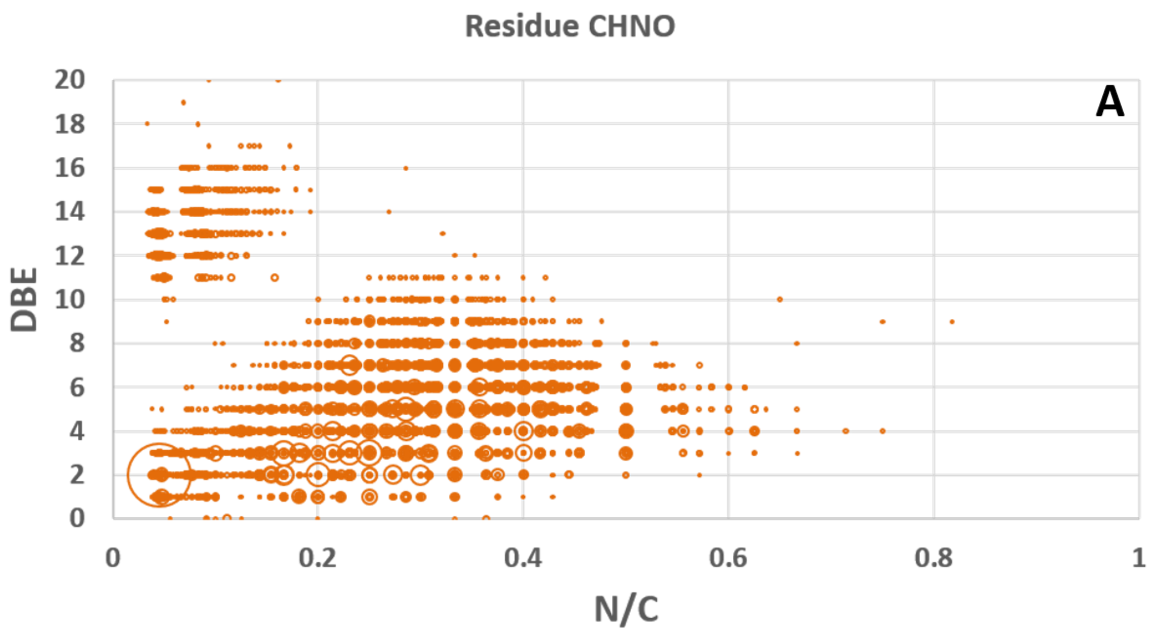


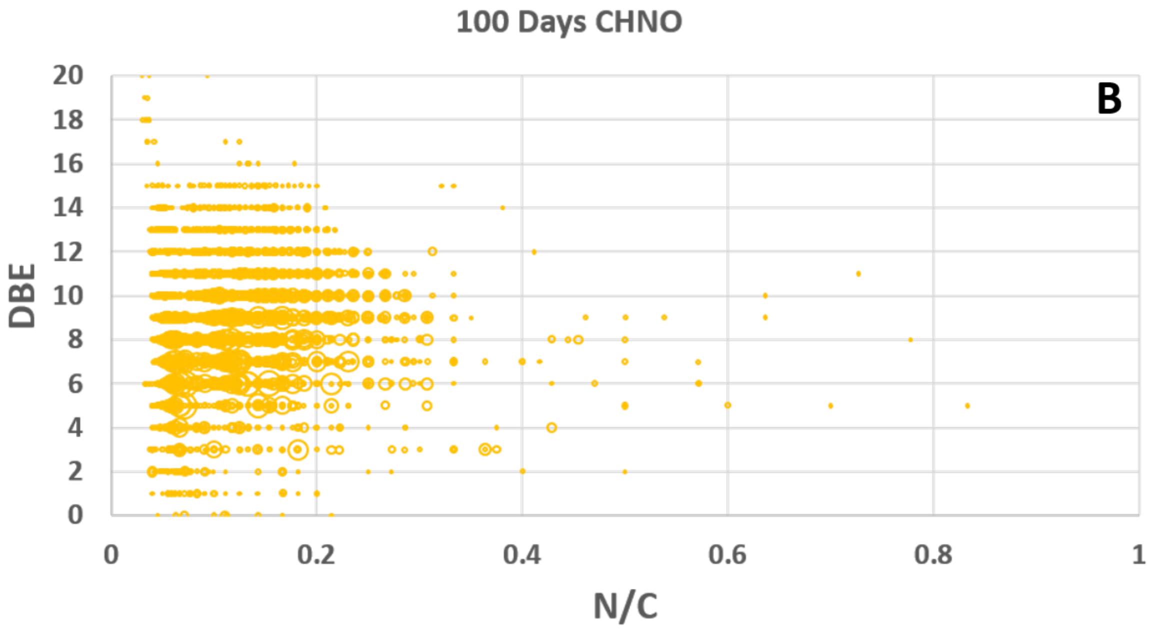


**Supplementary Figure 6– –** DBE vs N/C relative to the CHNO family for pre-accretional (3512 molecular attributions) (A) and post-aqueous organic products after 100 days (2591 molecular attributions) at 150°C (B). The size of the circle representing each molecular attribution is proportional to ion intensities.

**Complementary data regarding the O vs m/z of the CHNO family for pre-accretional and post-aqueous organic residues after 100 days of reaction**

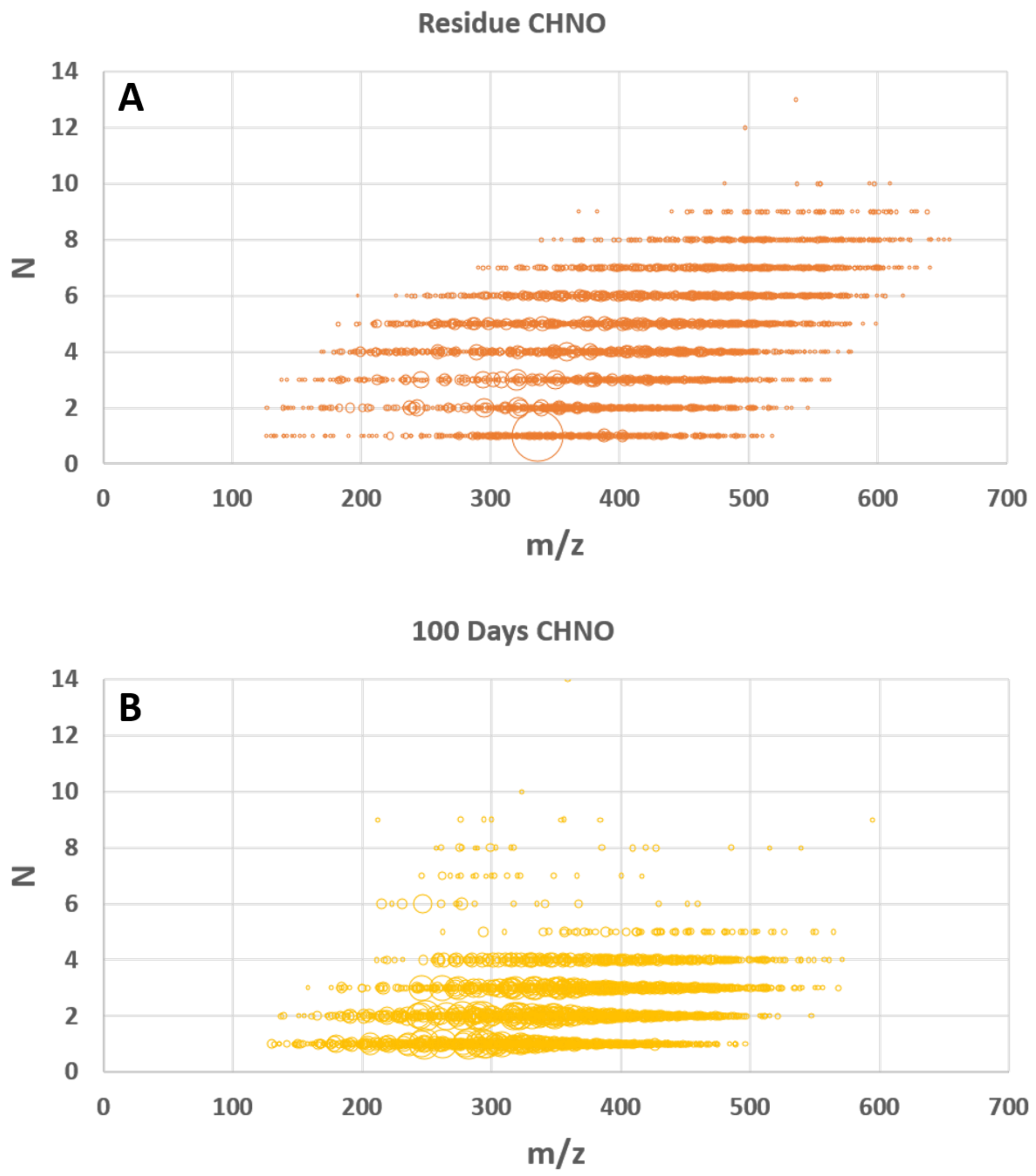


**Supplementary Figure 7 –** N vs m/z relative to the CHNO family for pre-accretional (3512 molecular attributions) (A) and post-aqueous organic products after 100 days (2591 molecular attributions) at 150°C (B). The size of the circle representing each molecular attribution is proportional to ion intensities.

**Complementary data regarding the Van Krevelen diagrams for H/C vs O/C of the CHNO family for pre-accretional and post-aqueous organic residues after 100 days of reaction**

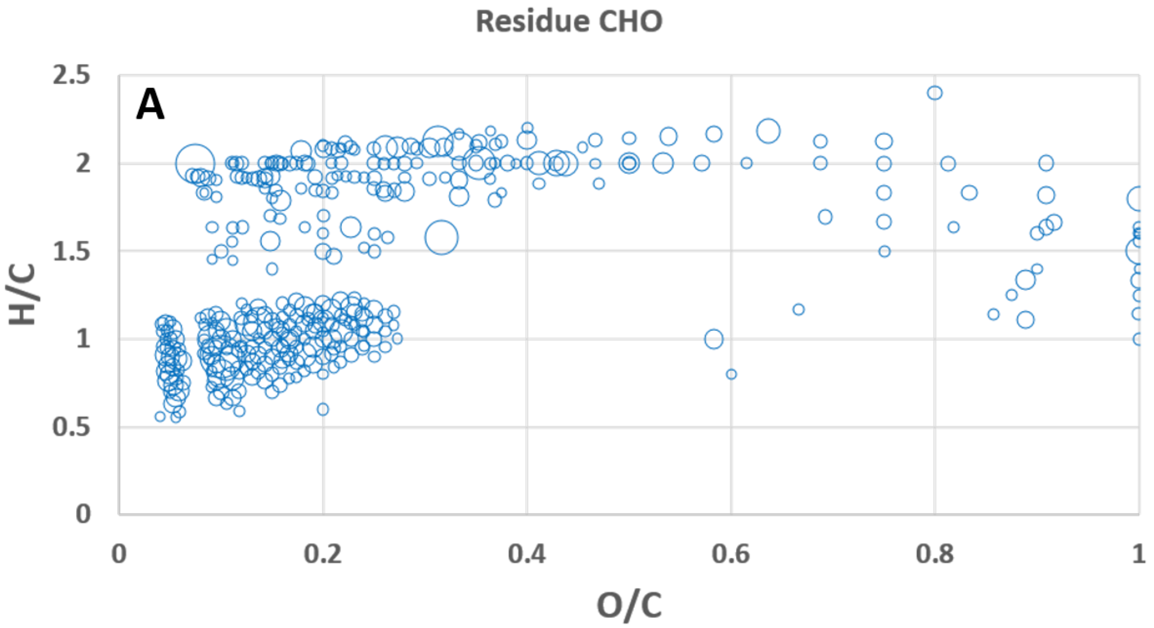


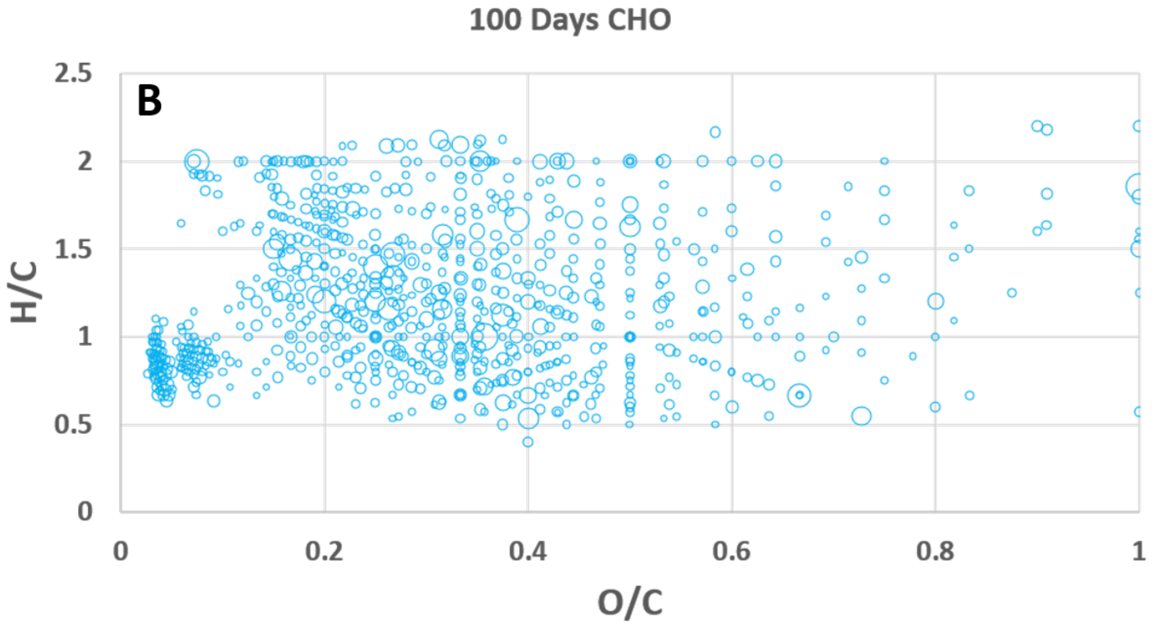


**Supplementary Figure 8 –** Van Krevelen diagrams are displayed for H/C vs O/C relative to the CHO family for pre-accretional (351 molecular attributions) (A) and post-aqueous organic products after 100 days (711 molecular attributions) at 150°C (B). The size of the circle representing each molecular attribution is proportional to ion intensities.

**Complementary data regarding the DBE vs O/C of the CHO family for pre-accretional and post-aqueous organic residues after 100 days of reaction**

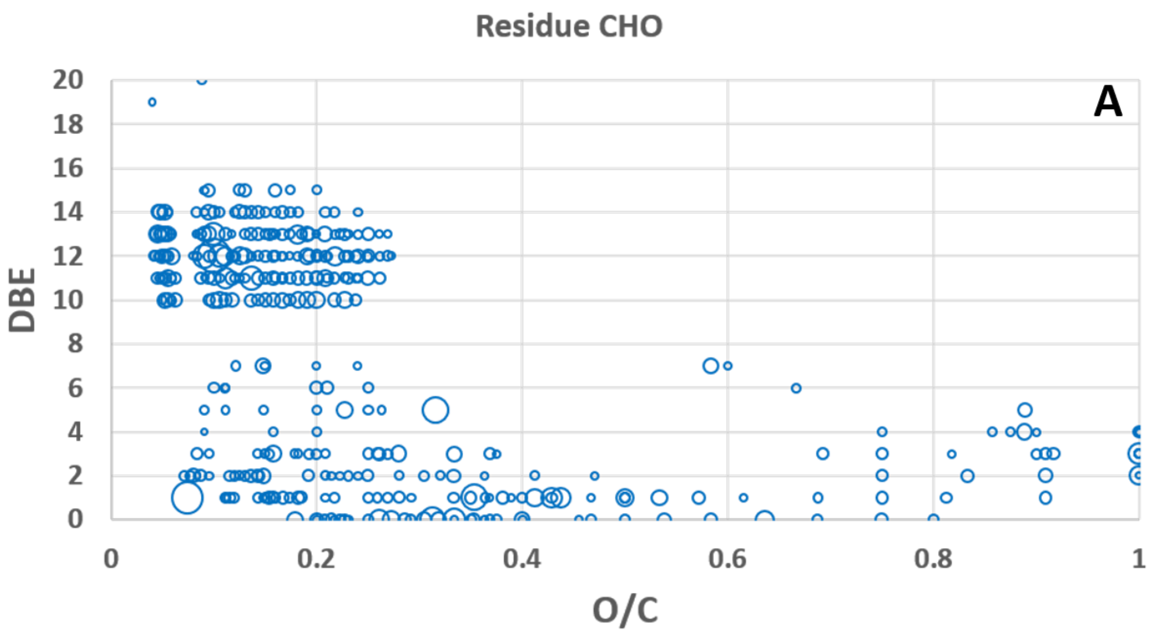


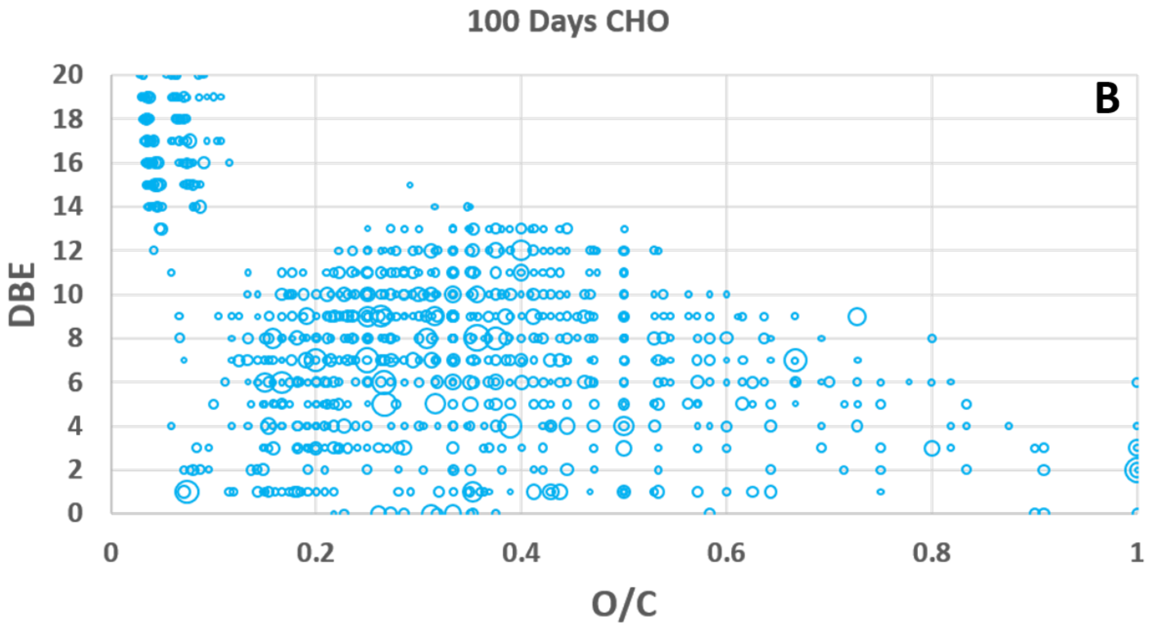


**Supplementary Figure 9–** DBE vs O/C relative to the CHO family for pre-accretional (351 molecular attributions) (A) and post-aqueous organic products after 100 days (711 molecular attributions) at 150°C (B). The size of the circle representing each molecular attribution is proportional to ion intensities.

**Complementary data regarding the O vs m/z of the CHO family for pre-accretional and post-aqueous organic residues after 100 days of reaction**

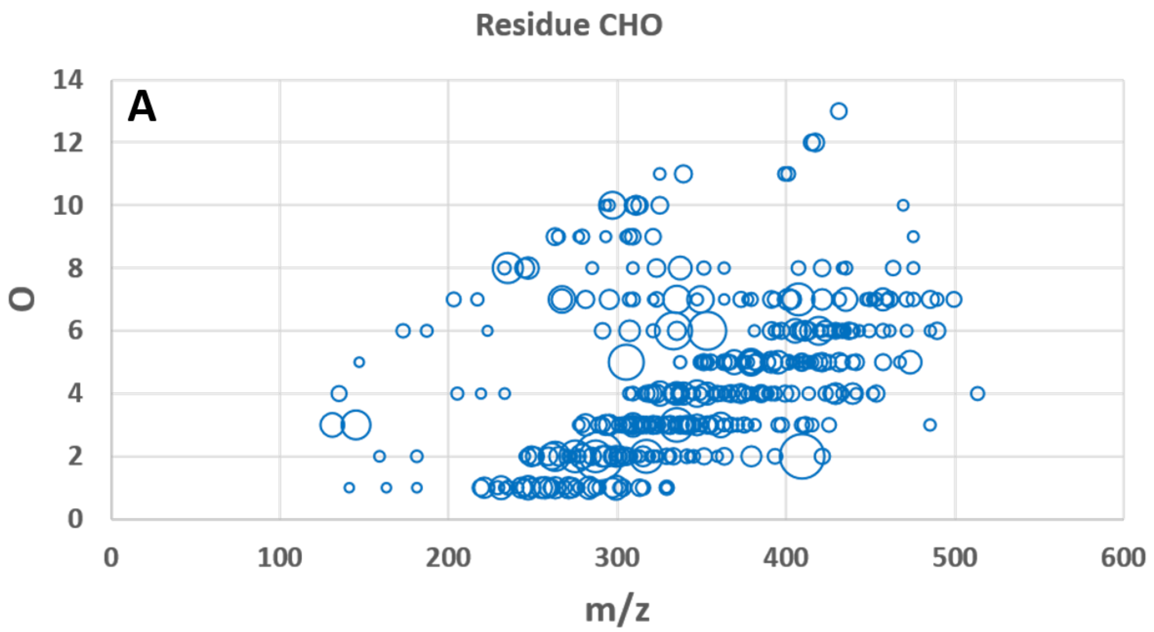


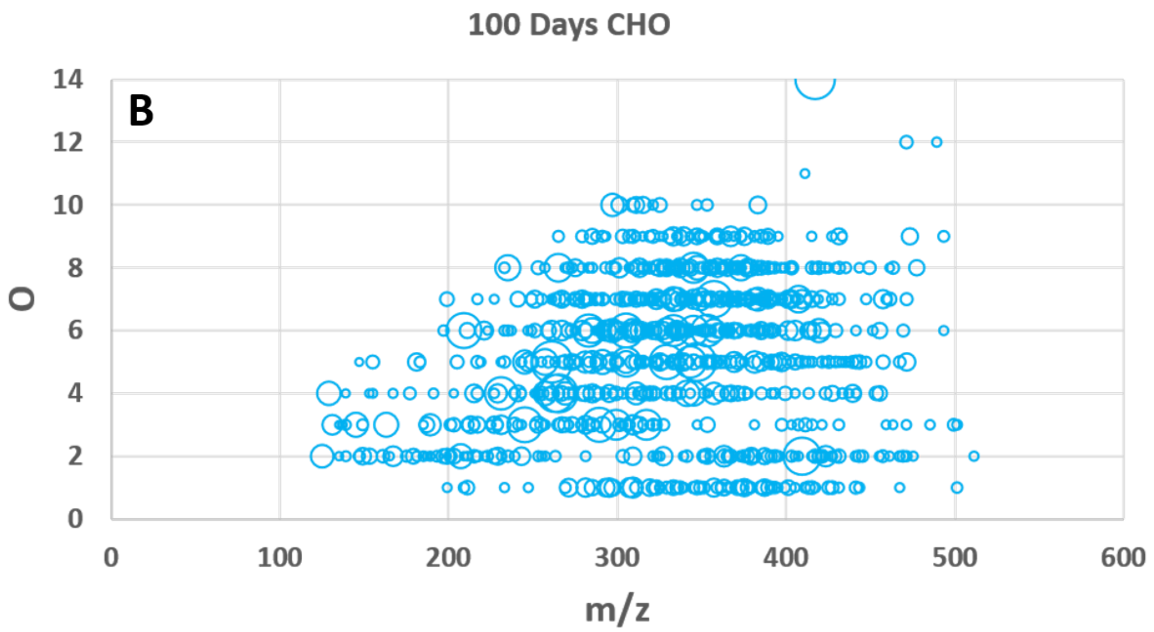


**Supplementary Figure 10 –** O vs m/z relative to the CHO family for pre-accretional (351 molecular attributions) (A) and post-aqueous organic products after 100 days (711 molecular attributions) at 150°C (B). The size of the circle representing each molecular attribution is proportional to ion intensities.

**Complementary data regarding the Van Krevelen diagrams for H/C vs N/C of the CHN family for pre-accretional and post-aqueous organic residues after 100 days of reaction**

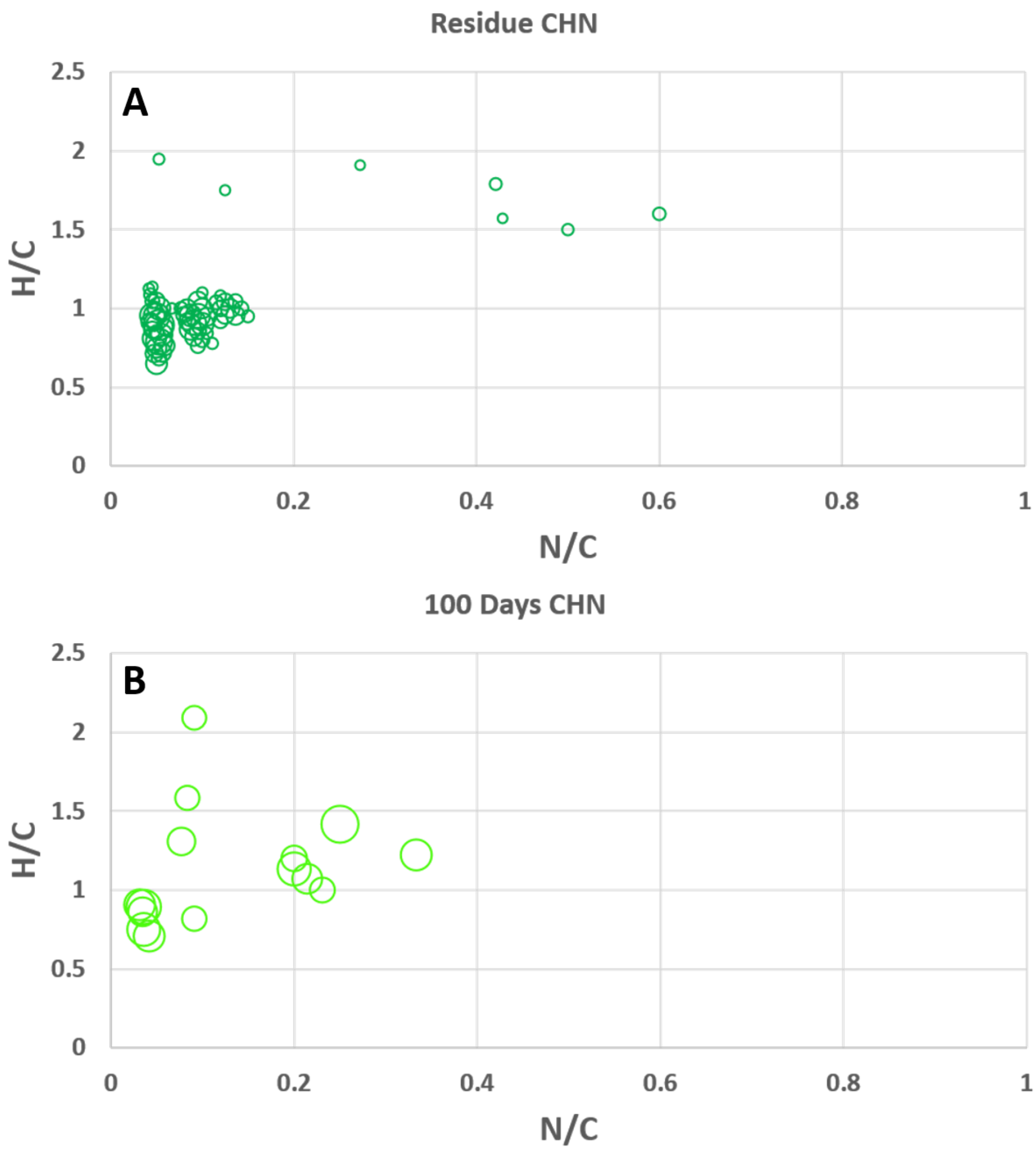


**Supplementary Figure 11 –** Van Krevelen diagrams are displayed for H/C vs N/C relative to the CHN family for pre-accretional (63 molecular attributions) (A) and post-aqueous organic products after 100 days (15 molecular attributions) at 150°C (B). The size of the circle representing each molecular attribution is proportional to ion intensities.

**Complementary data regarding the DBE vs N/C of the CHN family for pre-accretional and post-aqueous organic residues after 100 days of reaction**

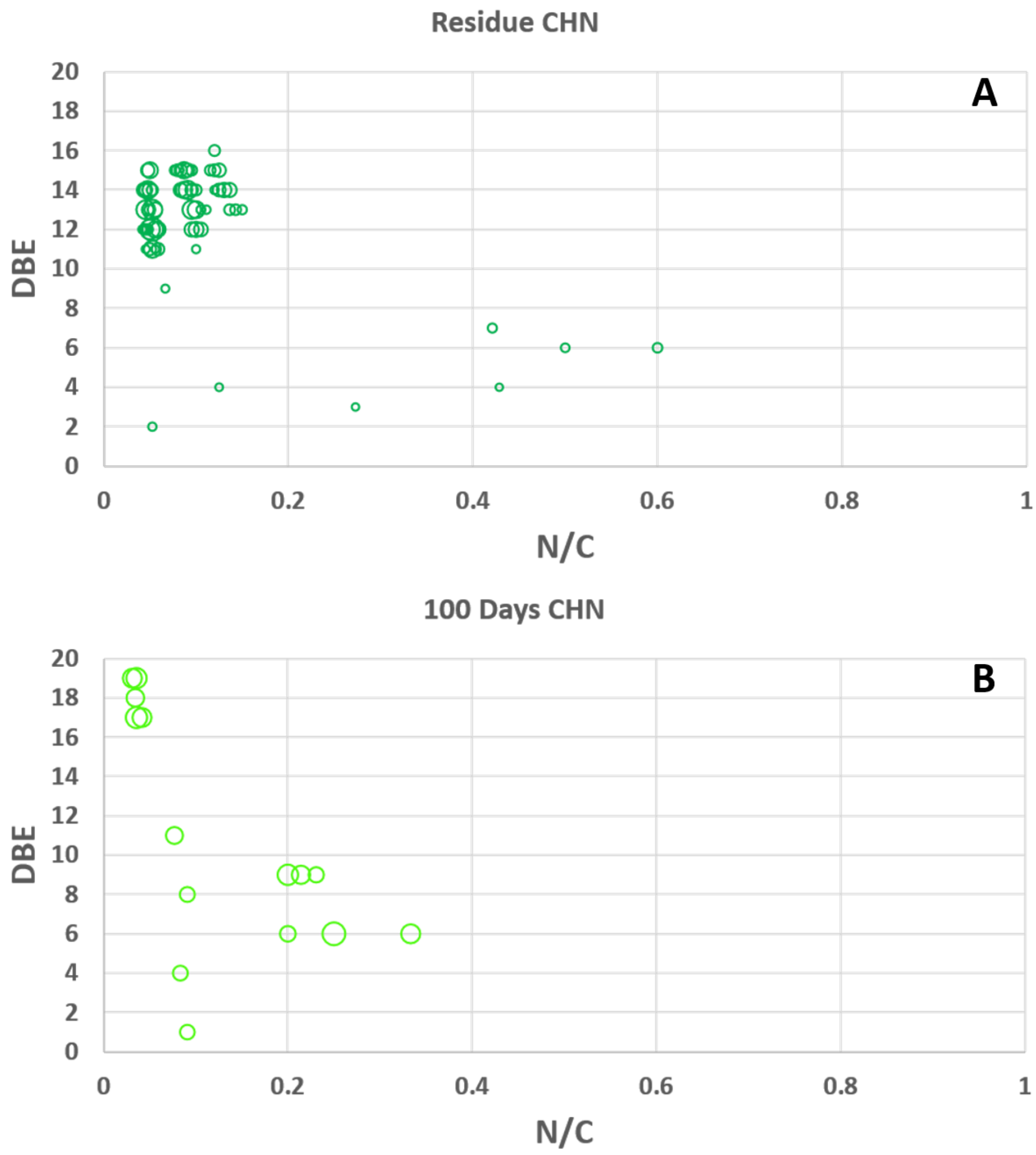


**Supplementary Figure 12–** DBE vs N/C relative to the CHN family for pre-accretional (63 molecular attributions) (A) and post-aqueous organic products after 100 days (15 molecular attributions) at 150°C (B). The size of the circle representing each molecular attribution is proportional to ion intensities.

**Complementary data regarding the N vs m/z of the CHN family for pre-accretional and post-aqueous organic residues after 100 days of reaction**

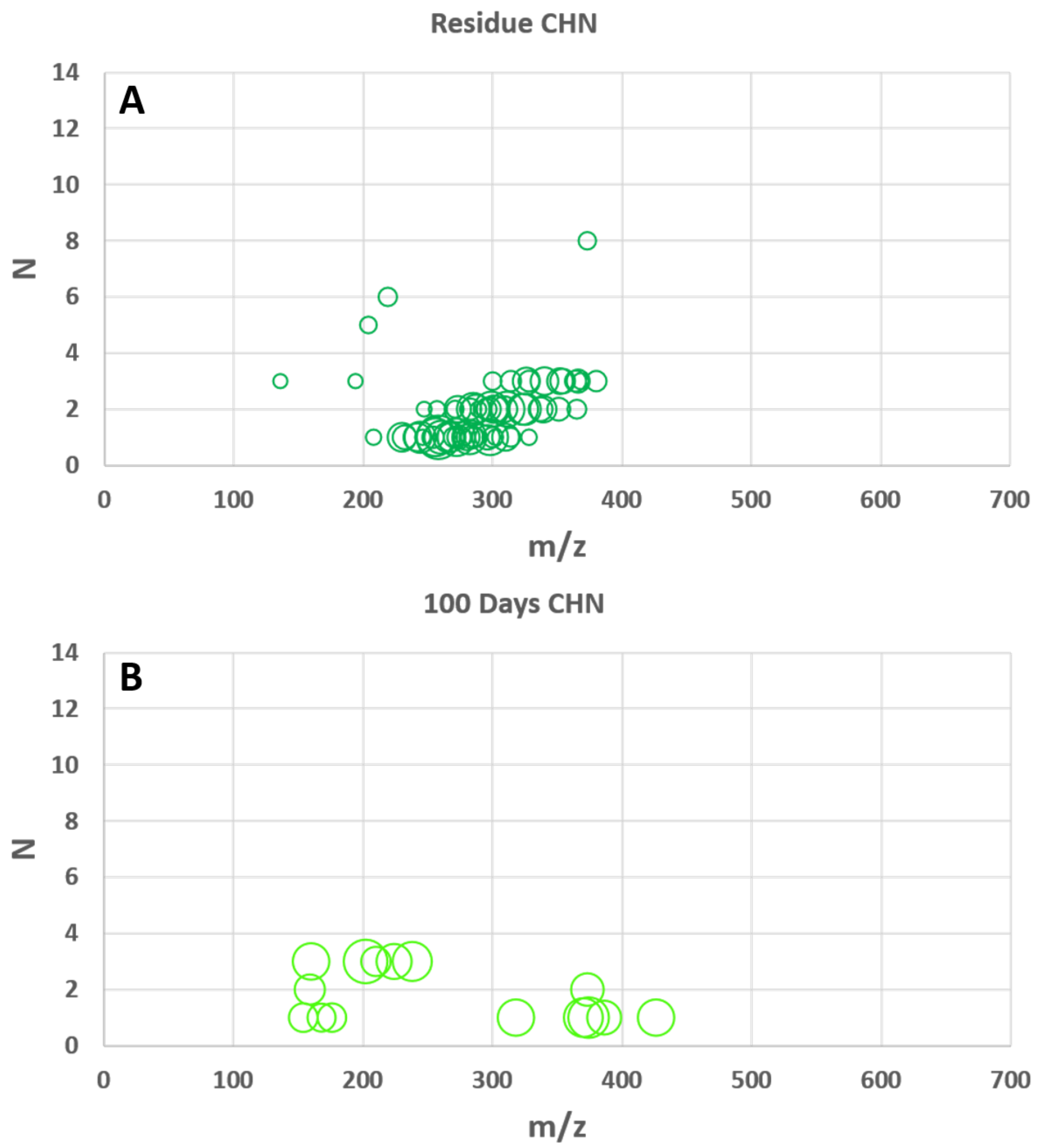


**Supplementary Figure 13 –** N vs m/z relative to the CHN family for pre-accretional (63 molecular attributions) (A) and post-aqueous organic products after 100 days (15 molecular attributions) at 150°C (B). The size of the circle representing each molecular attribution is proportional to ion intensities.

**Evolution of common molecular attributions between the different experimental times**

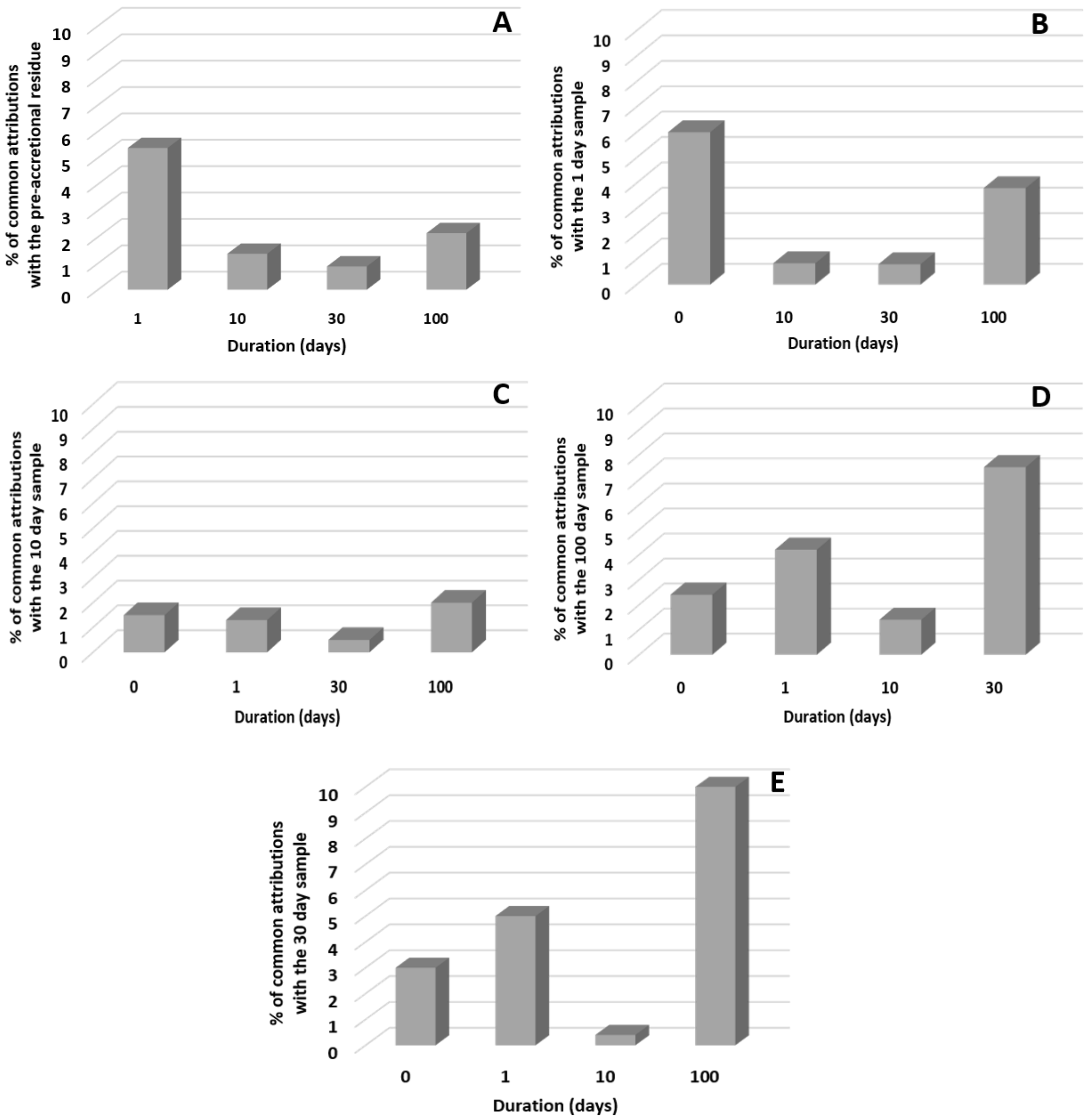


**Supplementary Figure 14 –** Common molecular attributions between samples of different experimental times. (A) displayed the common attributions of the pre-accretional organic residue with 1, 10, 30 or 100 days of experiments at 150°C. (B) displayed the common attributions of the 1 day sample at 150°C with the pre-accretional organic residue (0), 10, 30 or 100 days of experiments at 150°C. (C) displayed the common attributions of the 10 day sample at 150°C with the pre-accretional organic residue (0), 1, 30 or 100 days of experiments at 150°C. (D) displayed the common attributions of the 100 day sample at 150°C with the pre-accretional organic residue (0), 1, 10 or 30 days of experiments at 150°C. (E) displayed the common attributions of the 30 day sample at 150°C with the pre-accretional organic residue (0), 1, 10 or 100 days of experiments at 150°C.

**Common molecular attributions between the pre-accretional organic residue and the Murchison SOM**

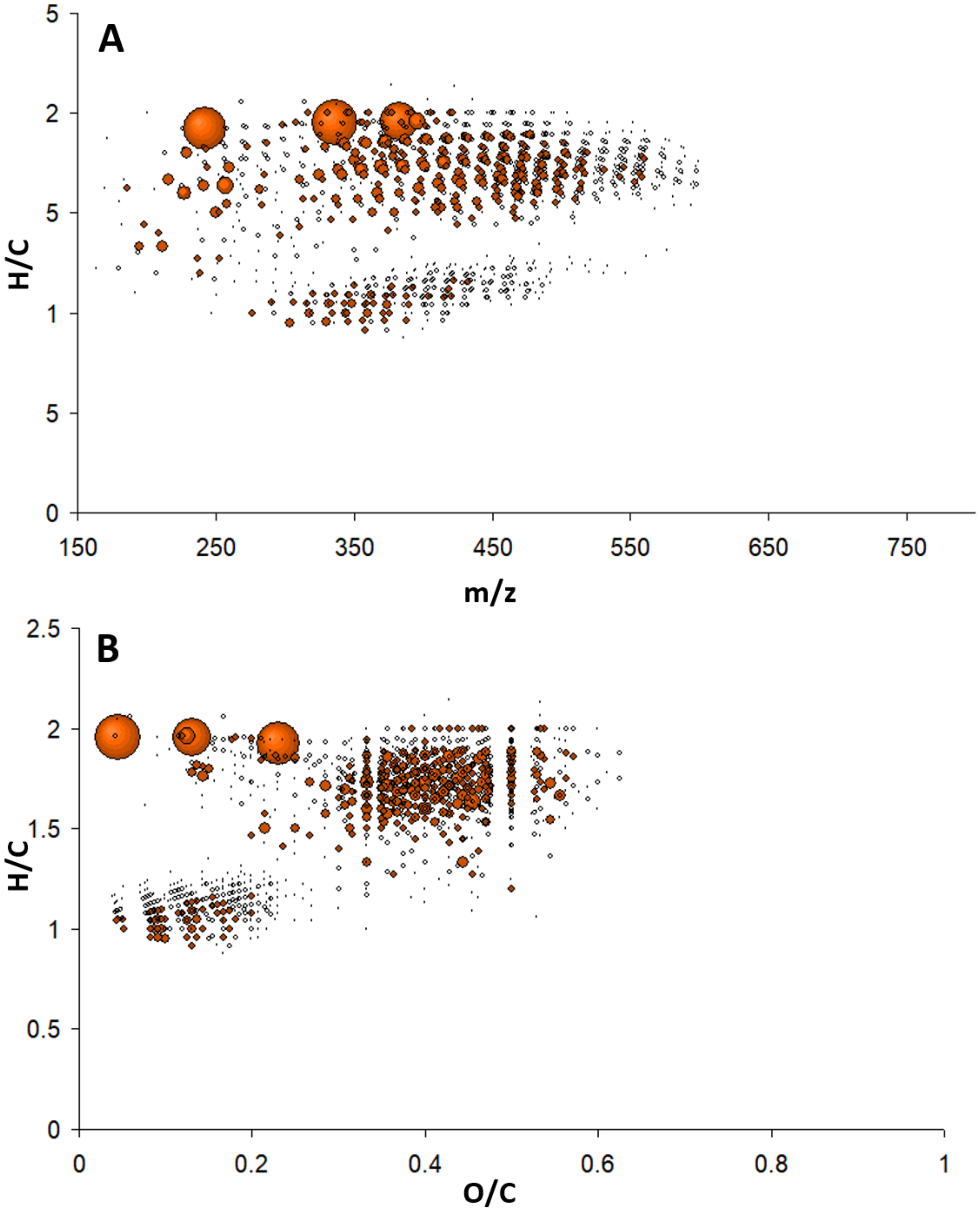


**Supplementary Figure 15 –** The 1000 molecular attributions in common between the pre-accretional organic residue and the SOM of Murchison represented as H/C vs m/z (A) or with the Van Krevelen diagram H/C vs O/C (B). The size of the circle representing each molecular attribution is proportional to ion intensities.

**Common molecular attributions between the post-accretional organic products and the Murchison SOM**

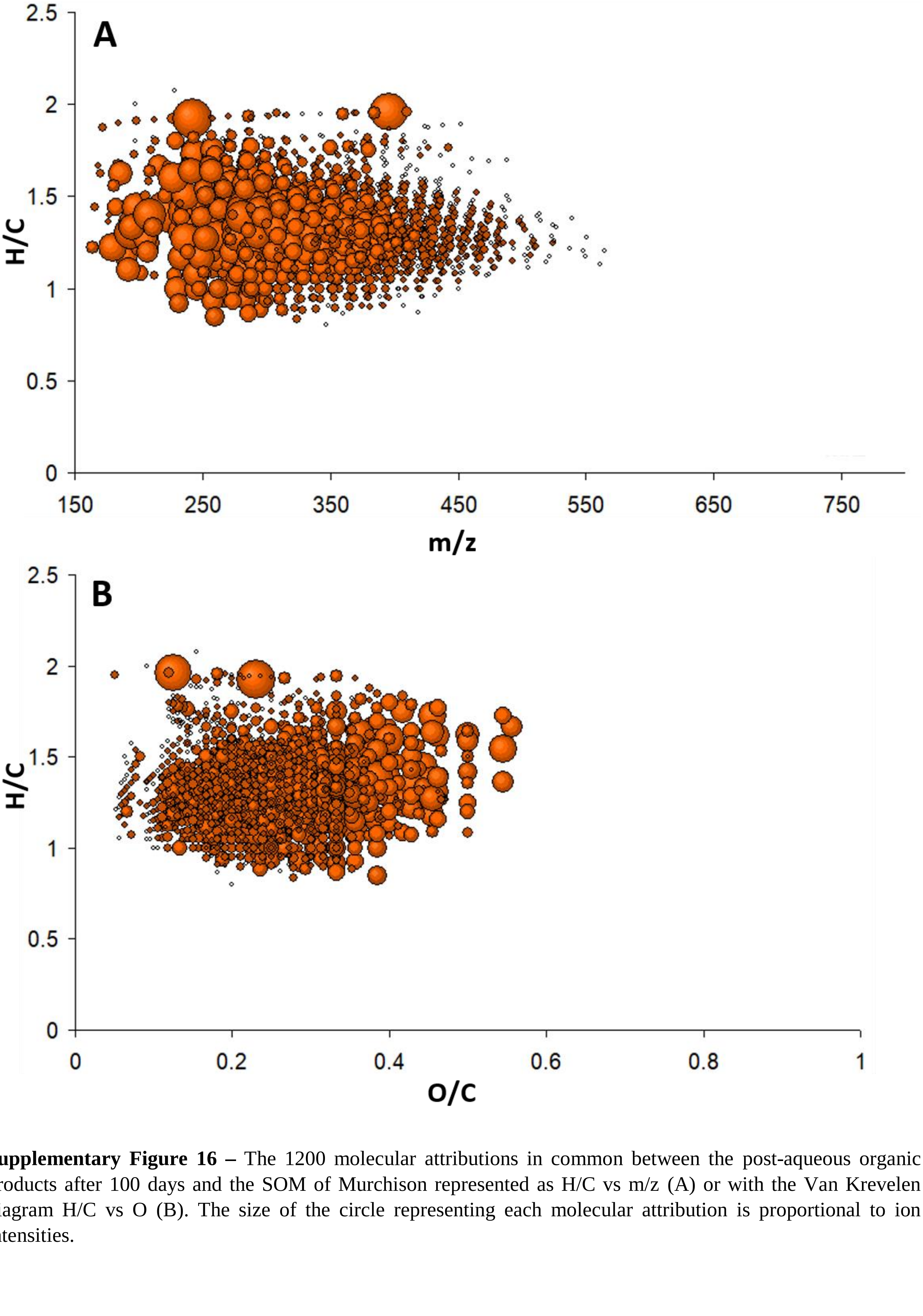


**Supplementary Figure 16 –** The 1200 molecular attributions in common between the post-aqueous organic products after 100 days and the SOM of Murchison represented as H/C vs m/z (A) or with the Van Krevelen diagram H/C vs O (B). The size of the circle representing each molecular attribution is proportional to ion intensities.

**Common molecular attributions between the pre-accretional organic residue, post-accretional organic products and the Murchison SOM**

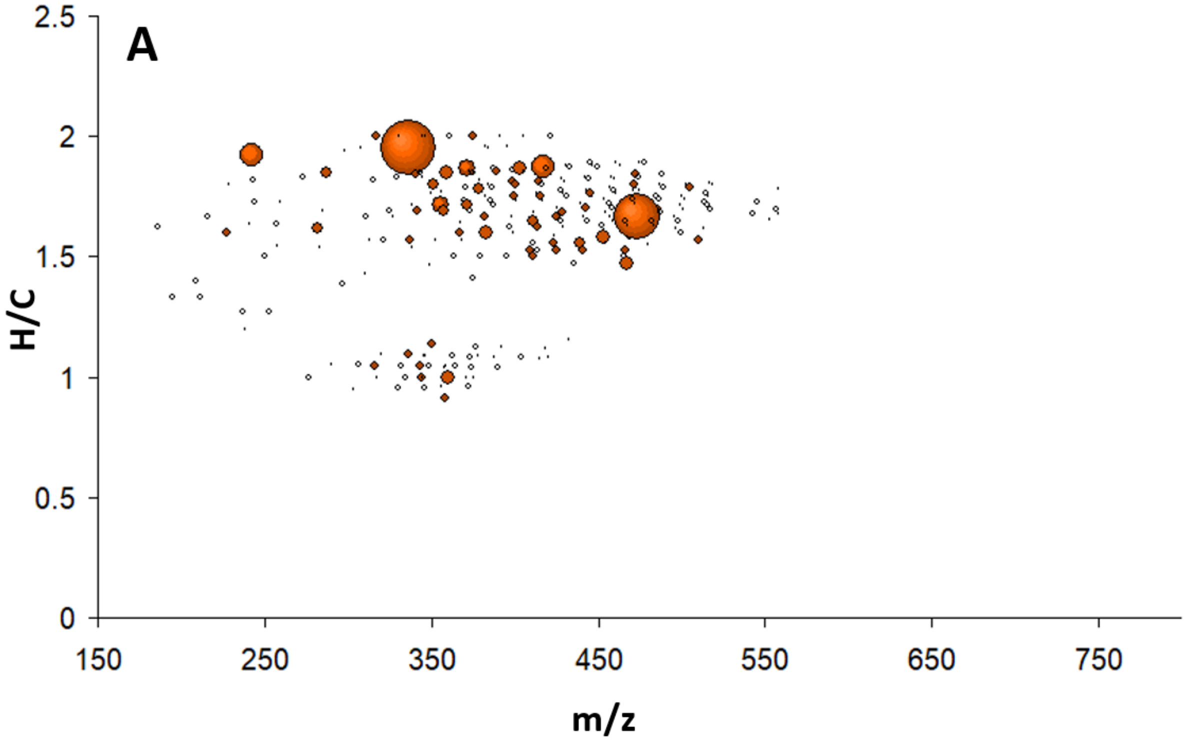


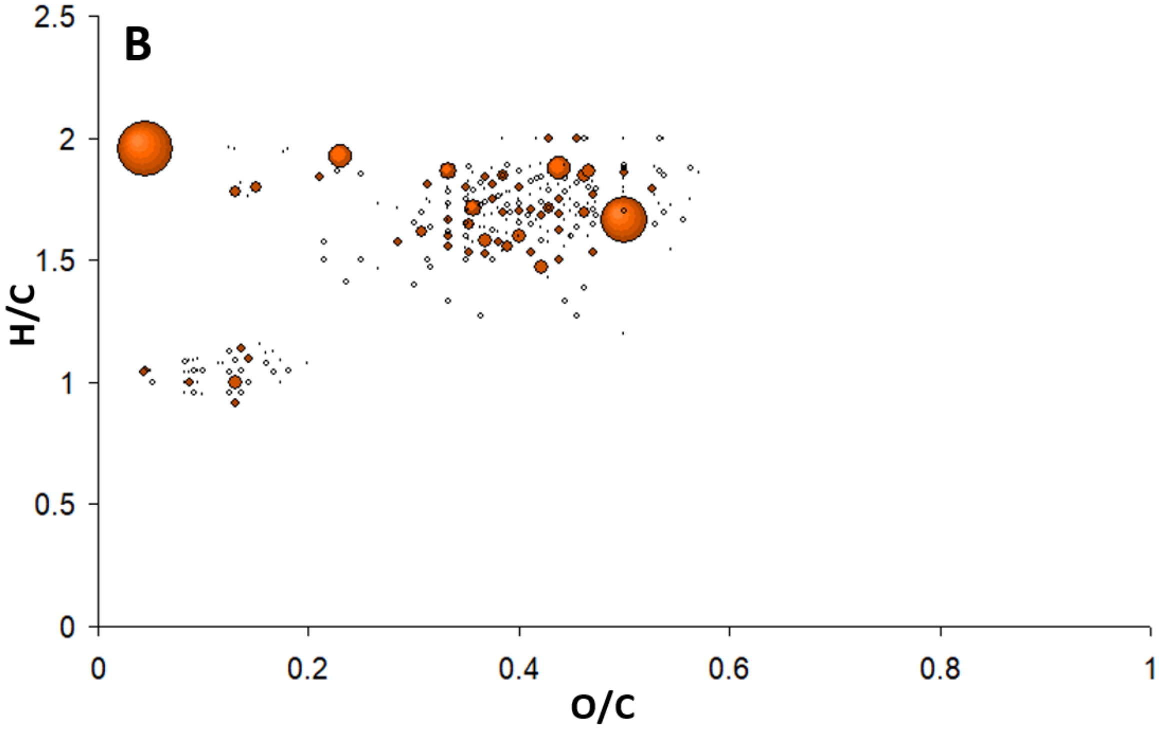


**Supplementary Figure 17 –** The 265 molecular attributions in common between pre-accretional residue, post-aqueous organic products after 100 days and the SOM of Murchison represented as H/C vs m/z (A) or with the Van Krevelen diagram H/C vs O/C (B). The size of the circle representing each molecular attribution is proportional to ion intensities.
